# Supplementary material for: PHAROS: turning single-cell perturbation models into target-directed drug-combination screens
Source: bioRxiv. 2026 Sep 10:2026.09.08.749477. Preprint. [Version 1] doi: 10.64898/2026.09.08.749477 (PMC13622985; doi:10.64898/2026.09.08.749477)
Supplement: Supplementary file 1 [file NIHPP2026.09.08.749477V1-supplement-1.pdf]

bioRxiv preprint doi: https://doi.org/10.64898/2026.09.08.749477; this version posted September 10, 2026. The copyright holder for this preprint (which was not certified by peer review) is the author/funder, who has granted bioRxiv a license to display the preprint in perpetuity. It is made available under a CC-BY 4.0 International license.

Supplementary Information

PHAROS: Turning single-cell perturbation models into target-directed drug-combination screens

bioRxiv preprint doi: https://doi.org/10.64898/2026.09.08.749477; this version posted September 10, 2026. The copyright holder for this preprint (which was not certified by peer review) is the author/funder, who has granted bioRxiv a license to display the preprint in perpetuity. It is made available under a CC-BY 4.0 International license.

S1 Supplementary Figures

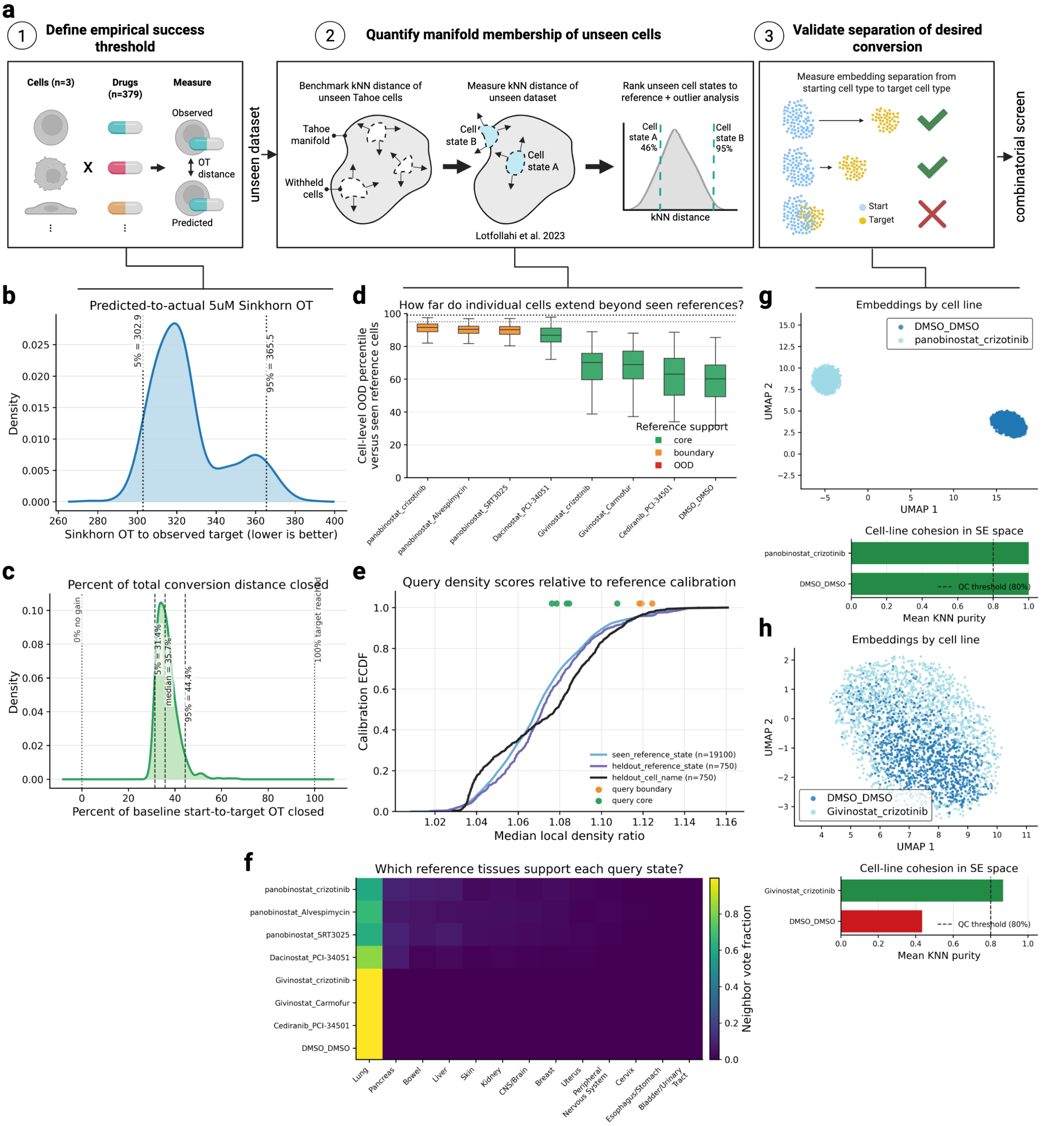

Figure S1: Admissibility framework a, Schematic of 3-part admissibility framework for unseen data. **b,** Distribution of sinkhorn OT distance from predicted single drug perturbation to observed perturbations across 3 cell lines and all 379 drugs at 5uM within Tahoe-100M. **c,** Distribution of source-to-target OT distance closed across 3 cell lines and all 379 drugs at 5uM within Tahoe-100M. **d,** Cell-level manifold percentiles relative to withheld Tahoe-100M cells across eight, 2-drug perturbations from unseen A549 data. Query states categorized into core (green), boundary (yellow), and out of distribution (red). **e,** Empirical cumulative distributions show median local-density ratios for seen, withheld-state, and withheld-cell-line Tahoe-100M calibrations. Larger values indicate weaker reference support. Query states are plotted above the distributions and colored by their support classification.

bioRxiv preprint doi: https://doi.org/10.64898/2026.09.08.749477; this version posted September 10, 2026. The copyright holder for this preprint (which was not certified by peer review) is the author/funder, who has granted bioRxiv a license to display the preprint in perpetuity. It is made available under a CC-BY 4.0 International license.

Figure S1 continued: f, Distance-weighted tissue annotations of the nearest Tahoe-100M reference neighbors for each query state. Rows denote query states, columns denote reference tissues and color indicates the fraction of total neighbor weight assigned to each tissue. **g,** An example of a specific conversion from start (DMSO) to target (panobinostat + crizotinib) that passed separation. Top: UMAP in SE embedding space. Bottom: barplot of mean KNN purity per cell state. **h,** An example of a specific conversion from start (DMSO) to target (givinostat + crizotinib) that failed separation. Top: UMAP in SE embedding space. Bottom: barplot of mean KNN purity per cell state.

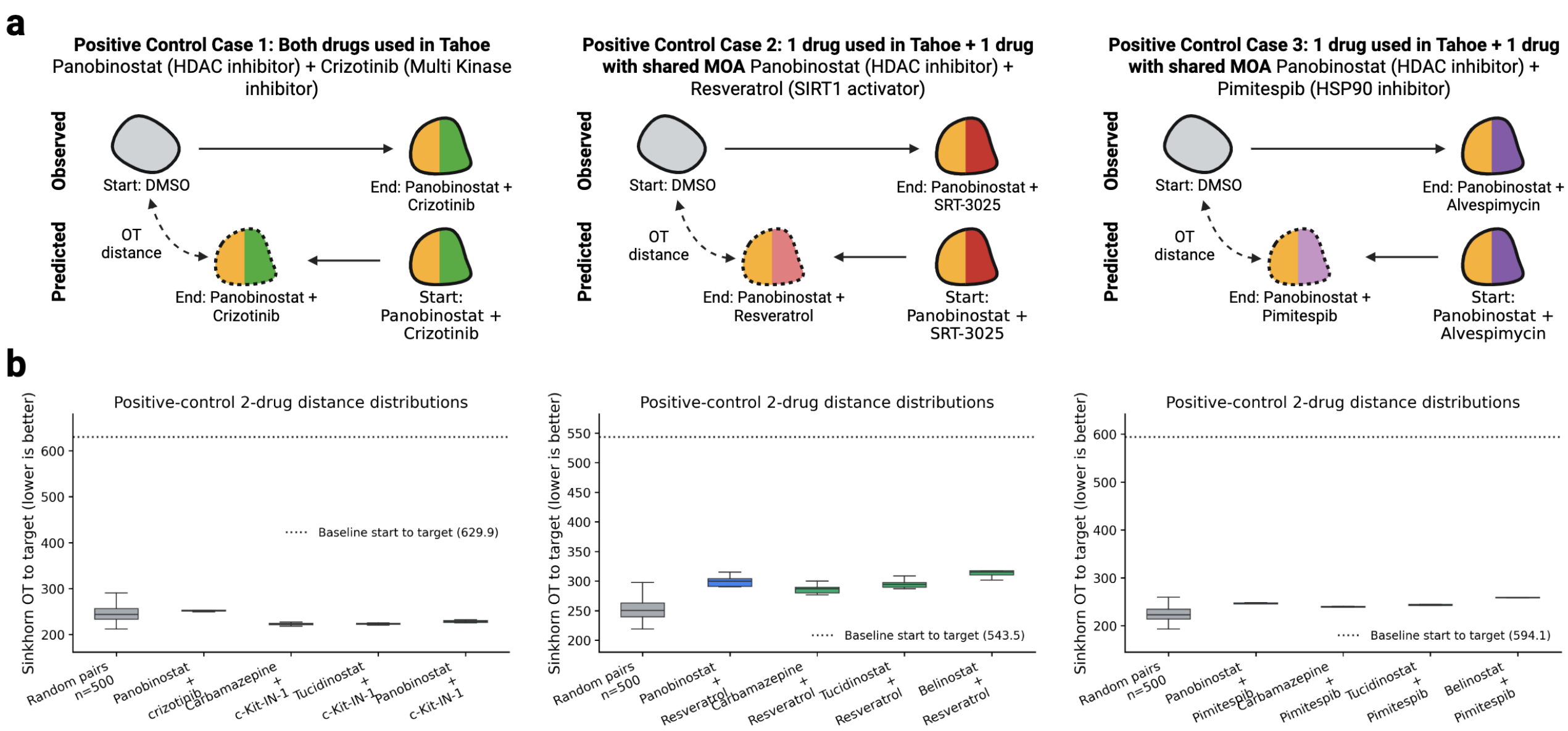

Figure S2: Efficacy when start and target are reversed. a, Schematic showing 3 positive control cases where the starting cell distribution and target cell distribution are reversed during prediction. In PHAROS the starting cell state is the DMSO no-drug state, the prediction is the change in latent space given the 2-drugs, and the prediction is compared against the experimentally observed 2-drug state. In this reversed orientation, the starting cell state is the experimentally observed 2-drug state, the prediction is the change in latent space given the 2-drugs, and the prediction is compared against the DMSO no-drug state. **b,** Distribution of sinkhorn OT distance to target under 2-drug predicted perturbations comparing 100 random pairs (grey), the exact 2-drug pair (blue), and all pairs with the same MOA (green). For each unordered drug pair, we evaluated all tested concentration combinations and both treatment orders, and retained the configuration with the lowest OT distance to the target. All 2-drugs were ran across 5 batches of starting and target cells. Dotted line is baseline OT distance between starting cell state and target cell state.

bioRxiv preprint doi: https://doi.org/10.64898/2026.09.08.749477; this version posted September 10, 2026. The copyright holder for this preprint (which was not certified by peer review) is the author/funder, who has granted bioRxiv a license to display the preprint in perpetuity. It is made available under a CC-BY 4.0 International license.

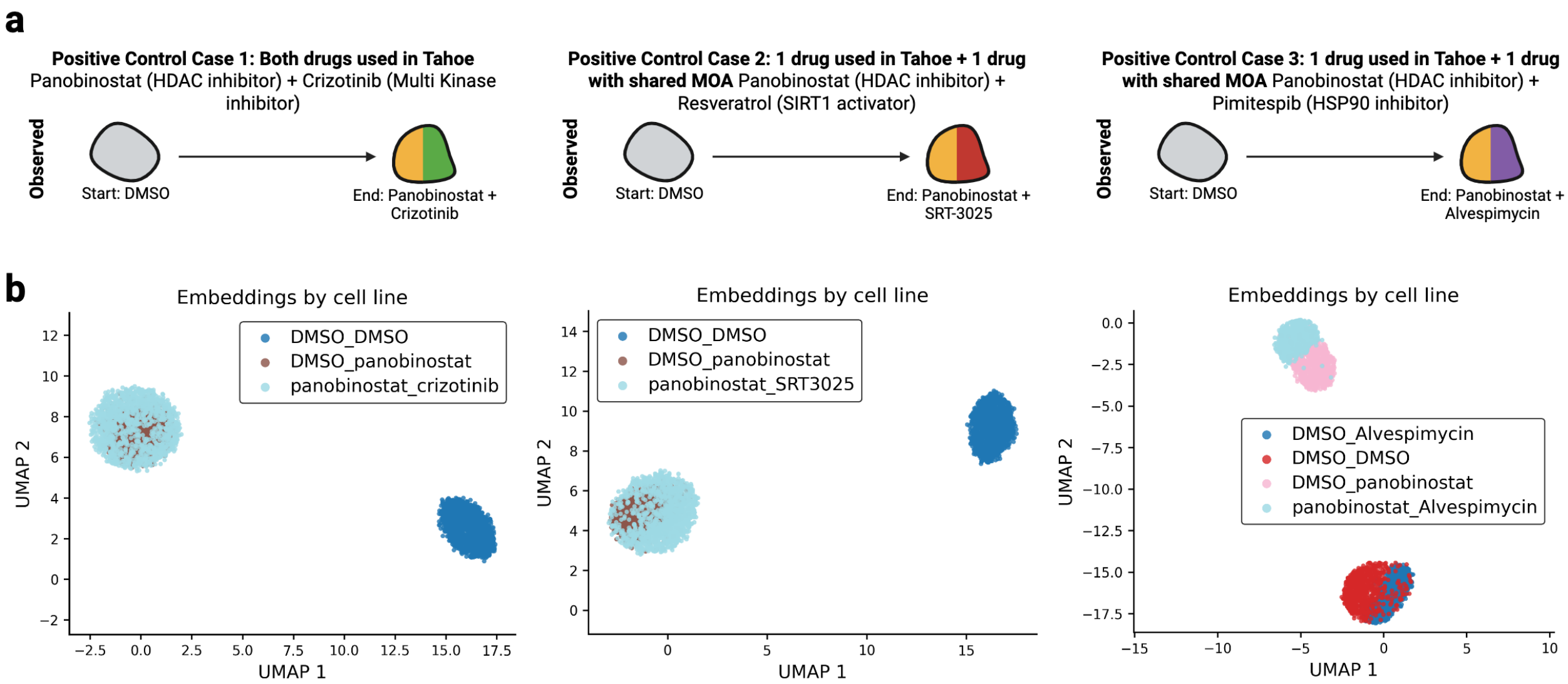

Figure S3: UMAP separation of experimental conditions in STATE single-cell foundation model embedding space. a, Schematic showing 3 positive control cases of 2-drug perturbations. **b,** UMAP visualization of experimentally measured cell states relevant to each of the 3 positive control cases. Case 1 and 2 contained just one of the single drug perturbations (DMSO + panobinostat), while case 3 contained both single drug perturbations.

bioRxiv preprint doi: https://doi.org/10.64898/2026.09.08.749477; this version posted September 10, 2026. The copyright holder for this preprint (which was not certified by peer review) is the author/funder, who has granted bioRxiv a license to display the preprint in perpetuity. It is made available under a CC-BY 4.0 International license.

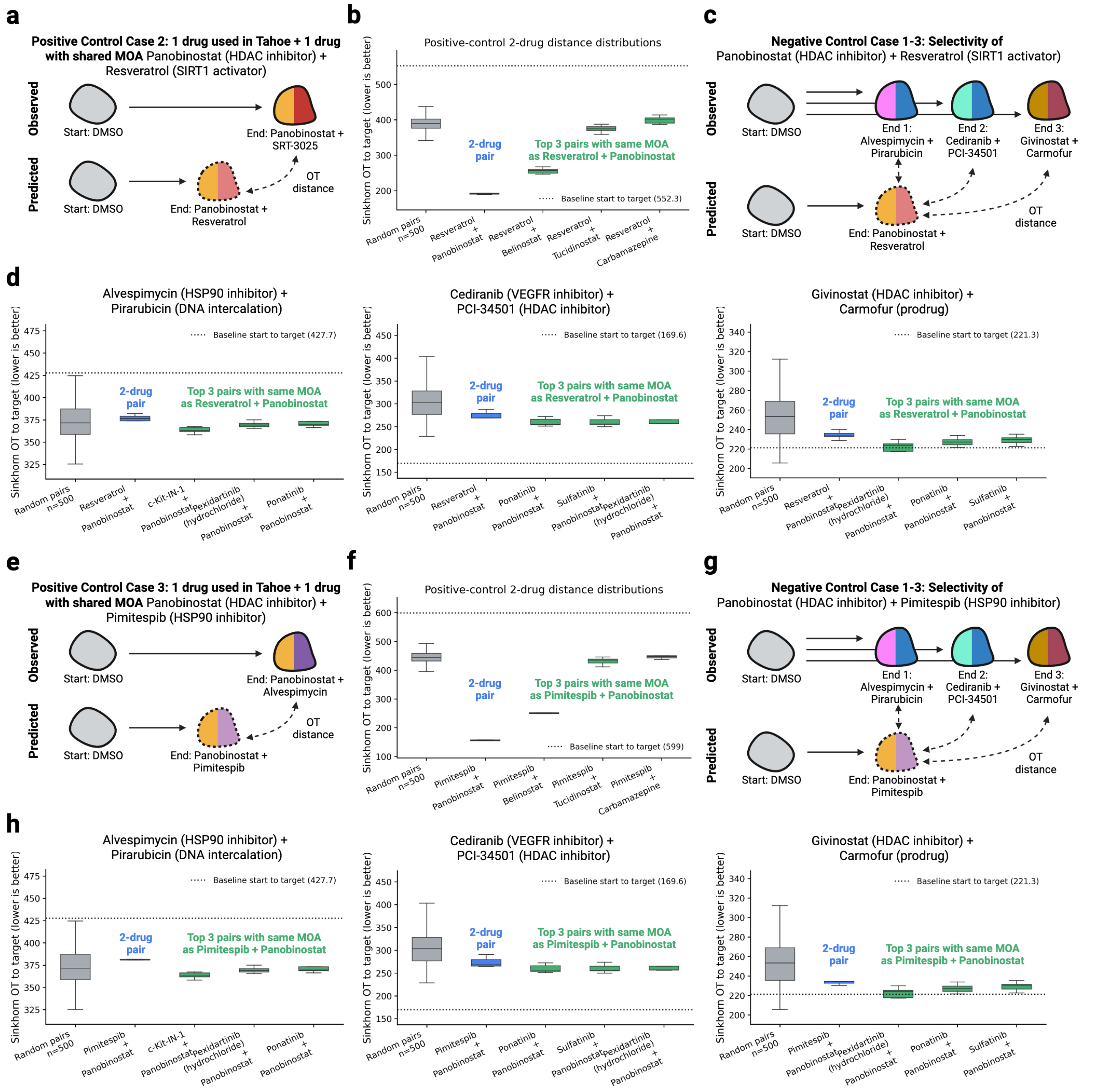

Figure S4: Specificity of positive control cases 2-3. **a,** Schematic of observed versus predicted for positive control case 2. **b,** Observed matches predicted. Distributions of OT distance to target comparing 100 random 2-drugs (grey), the panobinostat + resveratrol mechanism-matched proxy pair (blue), and top 3 pairs with matching MOA (green). **c,** Schematic demonstrating 3 counter-factual examples where the predicted pair, panobinostat + resveratrol, is compared against the observed pairs of alvespimycin + pirarubicin, cediranib + PCI34501, and givinostat + carmofur. **d,** 3 scenarios where observed does not match predicted. Distributions of OT distance to target comparing 100 random 2-drugs (grey), the panobinostat + resveratrol mechanism-matched proxy pair (blue), and top 3 pairs with matching MOA (green). **e,** Schematic of observed versus predicted for positive control case 3. **f,** Observed matches predicted. Distributions of OT distance to target comparing 100 random 2-drugs (grey), the panobinostat + pimitespib mechanism-matched proxy pair (blue), and top 3 pairs with matching MOA (green). **g,** Schematic demonstrating 3 counter-factual examples where the predicted pair, panobinostat + pimitespib, is compared against the observed pairs of alvespimycin + pirarubicin, cediranib + PCI34501, and givinostat + carmofur. **h,** 3 scenarios where observed does not match predicted. Distributions of OT distance to target comparing 100 random 2-drugs (grey), the panobinostat + pimitespib mechanism-matched proxy pair (blue), and top 3 pairs with matching MOA (green).

bioRxiv preprint doi: https://doi.org/10.64898/2026.09.08.749477; this version posted September 10, 2026. The copyright holder for this preprint (which was not certified by peer review) is the author/funder, who has granted bioRxiv a license to display the preprint in perpetuity. It is made available under a CC-BY 4.0 International license.

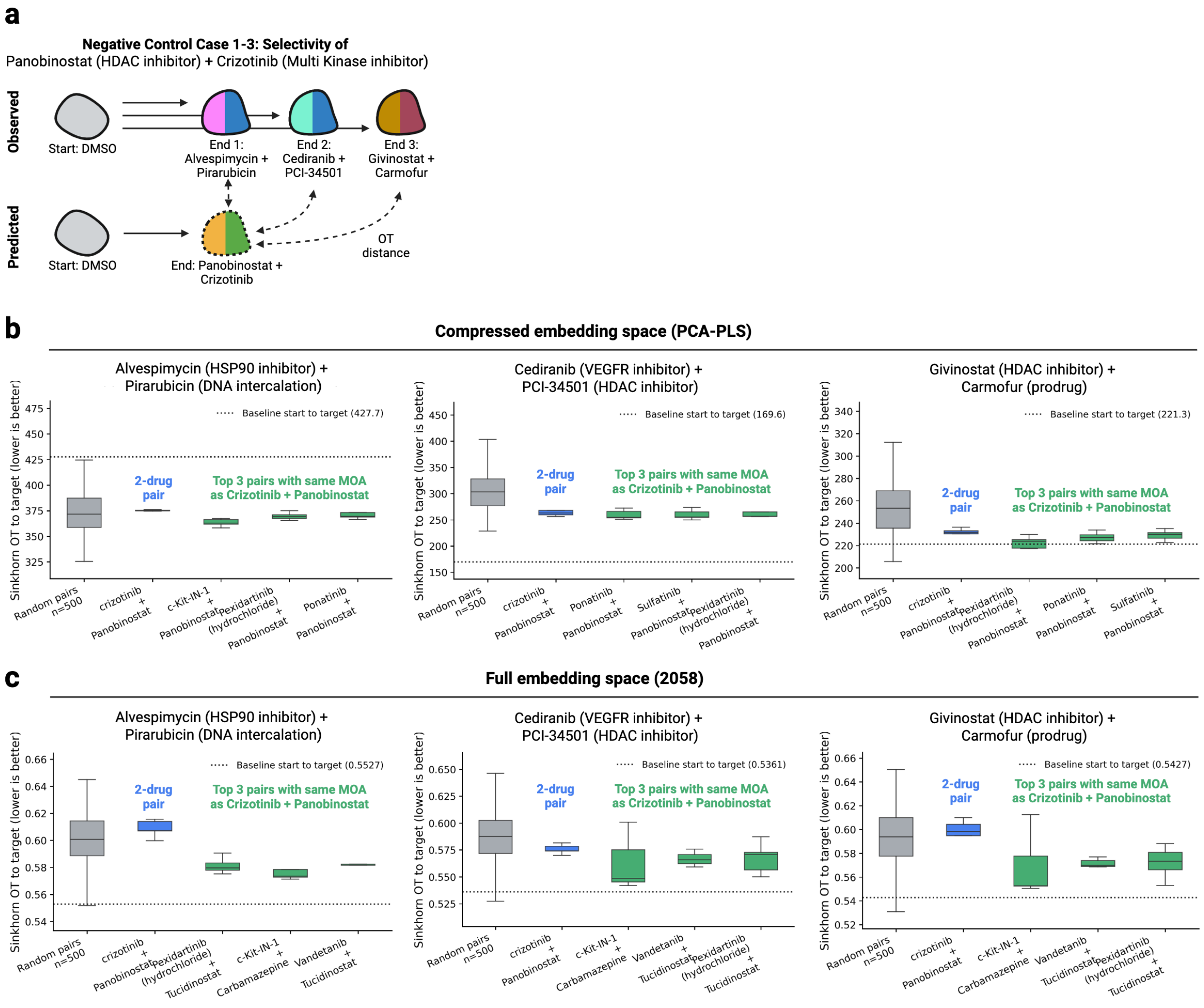

Figure S5: Effect of compression on specificity. **a,** Schematic demonstrating 3 counter-factual examples where the predicted pair, panobinostat + crizotinib, is compared against the observed pairs of alvespimycin + pirarubicin, cediranib + PCI34501, and givinostat + carmofur. **b,** Results shown in Fig 3d. Generated with default PCA-PLS compression. 3 scenarios where observed does not match predicted. Distributions of OT distance to target comparing 100 random 2-drugs (grey), exact panobinostat + crizotinib (blue), and top 3 pairs with matching MOA (green). All 2-drug predictions were evaluated across 5 batches of starting and target cells. **c,** 3 scenarios where observed does not match predicted. Generated without PCA-PLS compression, operating in full dimensionality (2058). Distributions of OT distance to target comparing 100 random 2-drugs (grey), exact panobinostat + crizotinib (blue), and top 3 pairs with matching MOA (green). All 2-drug predictions were evaluated across 5 batches of starting and target cells.

bioRxiv preprint doi: https://doi.org/10.64898/2026.09.08.749477; this version posted September 10, 2026. The copyright holder for this preprint (which was not certified by peer review) is the author/funder, who has granted bioRxiv a license to display the preprint in perpetuity. It is made available under a CC-BY 4.0 International license.

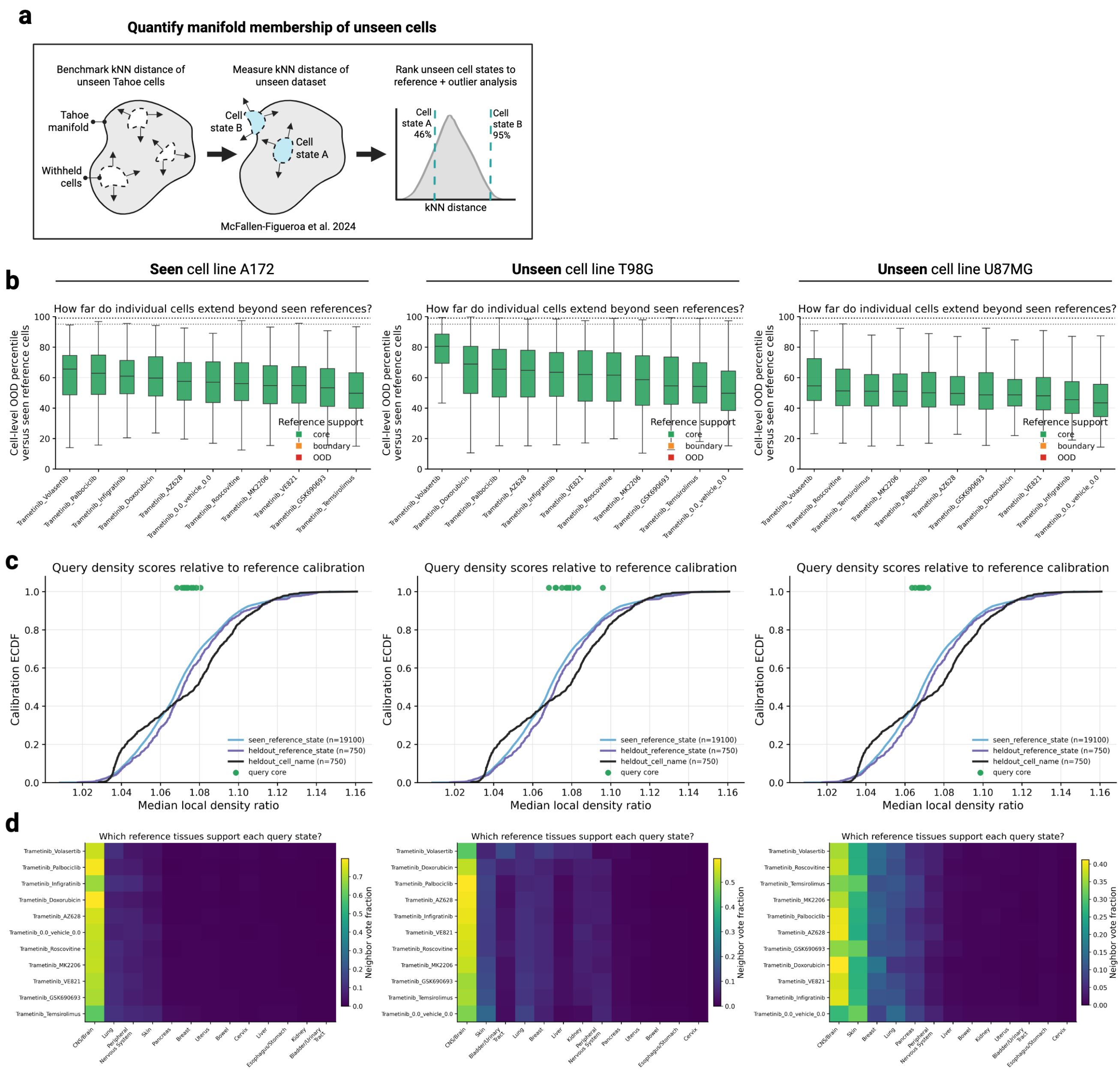

Figure S6: Manifold admissibility of 3 brain cancer cell lines. **a,** Part 2 of the admissibility framework. Quantify Tahoe-100M manifold membership of unseen cell states. **b-d,** Left: seen cell line A172. Middle: unseen cell line T98G. Right: unseen cell line U87MG. **b,** Cell-level manifold percentiles relative to withheld Tahoe-100M cells. Query states categorized into core (green), boundary (yellow), and out of distribution (red). **c,** Empirical cumulative distributions show median local-density ratios for seen, withheld-state, and withheld-cell-line Tahoe-100M calibrations. Larger values indicate weaker reference support. Query states are plotted above the distributions and colored by their support classification. **d,** Distance-weighted tissue annotations of the nearest Tahoe-100M reference neighbors for each query state. Rows denote query states, columns denote reference tissues and color indicates the fraction of total neighbor weight assigned to each tissue.

bioRxiv preprint doi: https://doi.org/10.64898/2026.09.08.749477; this version posted September 10, 2026. The copyright holder for this preprint (which was not certified by peer review) is the author/funder, who has granted bioRxiv a license to display the preprint in perpetuity. It is made available under a CC-BY 4.0 International license.

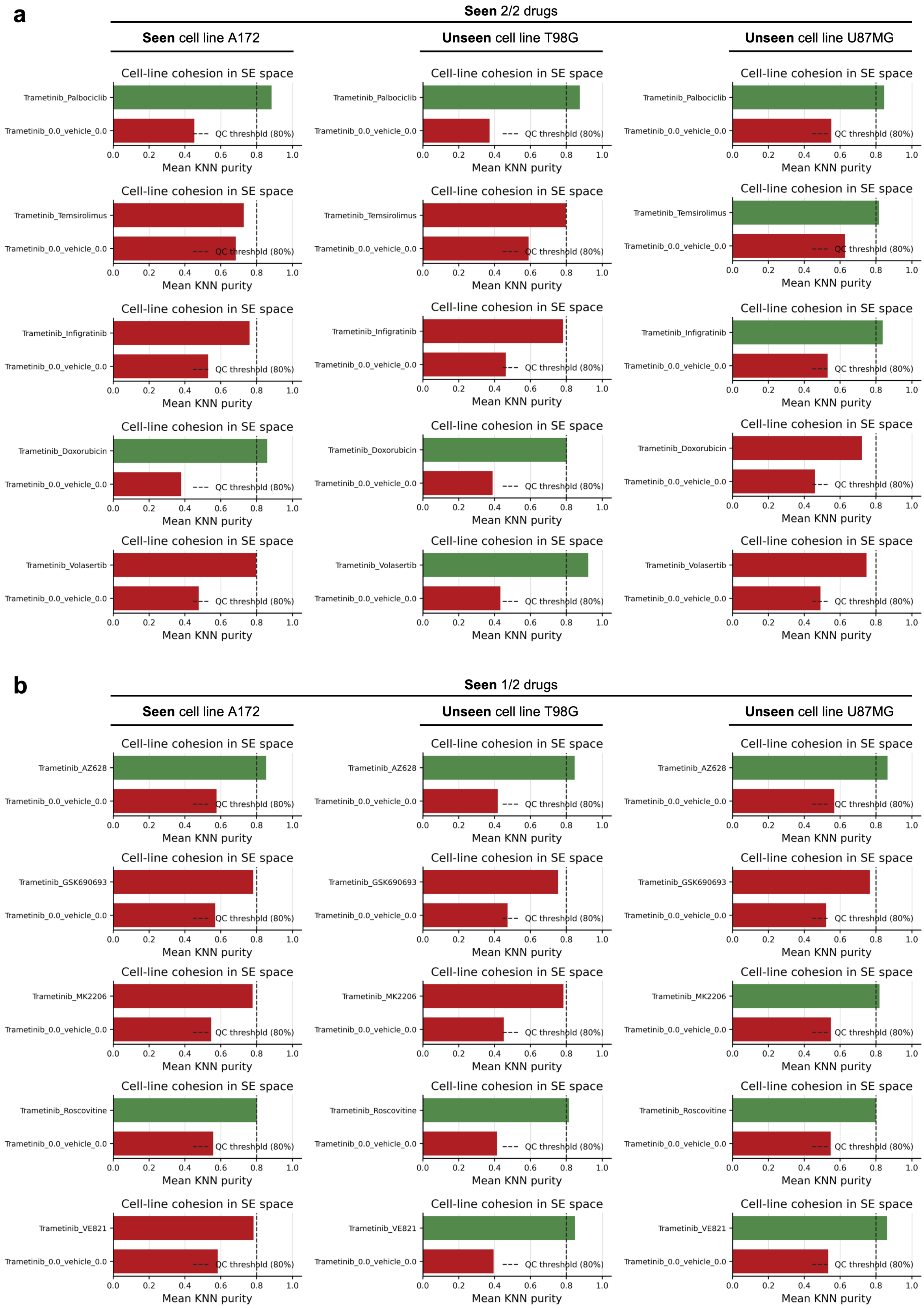

Figure S7: Source-target separation admissibility for every conversion within 3 brain cancer cell lines. a-b, Left: seen cell line A172. Middle: unseen cell line T98G. Right: unseen cell line U87MG. **a,** Part 3 of the admissibility framework for seen 2/2 drugs. Barplots of mean KNN purity per cell state for every conversion. **b,** Part 3 of the admissibility framework for seen 1/2 drugs. Barplots of mean KNN purity per cell state for every conversion.

bioRxiv preprint doi: https://doi.org/10.64898/2026.09.08.749477; this version posted September 10, 2026. The copyright holder for this preprint (which was not certified by peer review) is the author/funder, who has granted bioRxiv a license to display the preprint in perpetuity. It is made available under a CC-BY 4.0 International license.

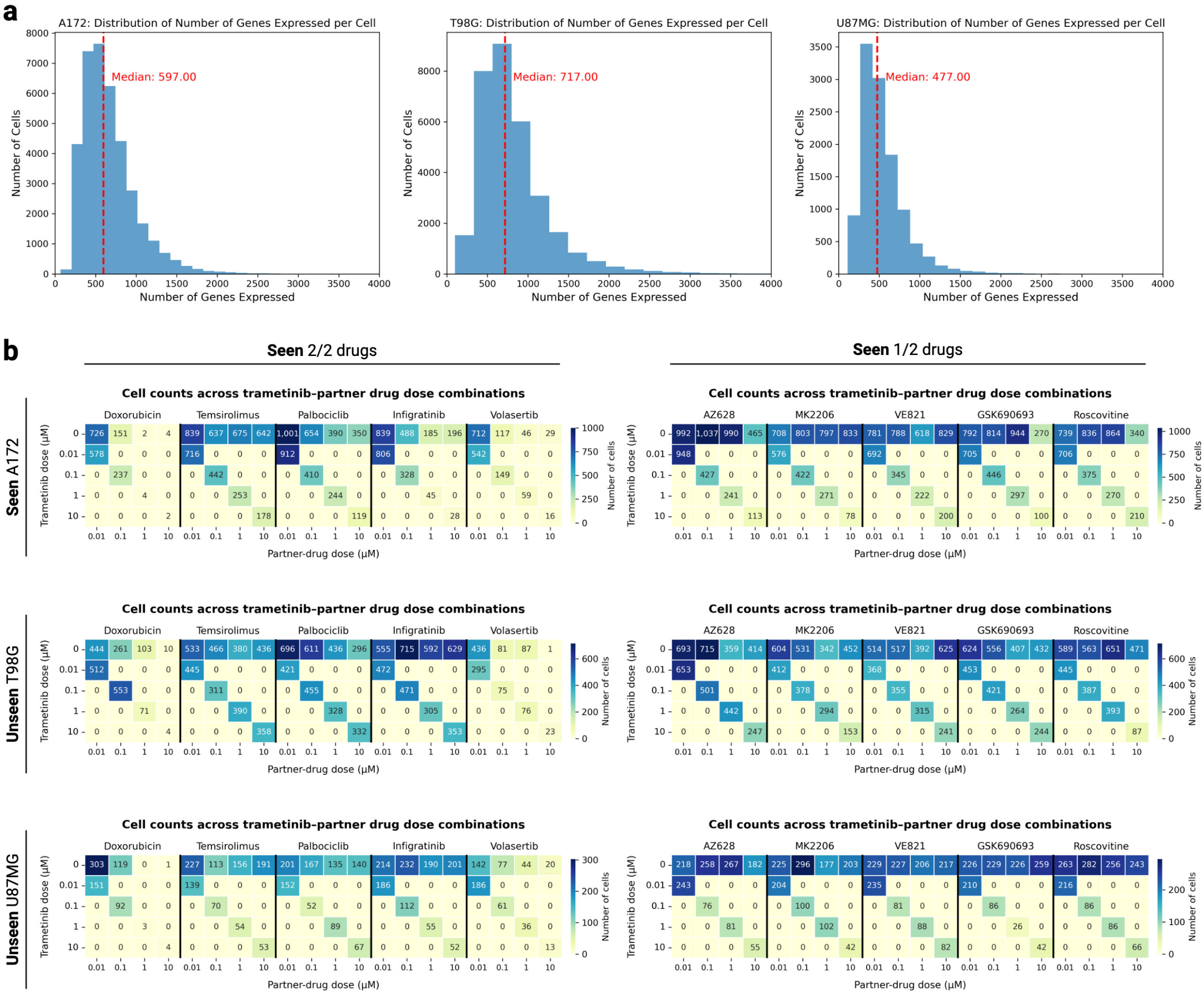

Figure S8: QC metrics for the 3 brain cancer cell lines. a, Distribution of genes captured per cell for seen A172 (left), unseen T98G (middle), and unseen U87MG (right). Red dotted line indicates median value. **b,** Left: seen 2/2 drugs. Right: seen 1/2 drugs. Top: seen cell line A172. Middle: unseen cell line T98G. Bottom: unseen cell line U87MG. Heatmap of total number of cells found in each 2-drug perturbation condition. Y-axis denotes the five concentrations (uM) of trametinib (0, 0.01, 0.1, 1, 10), which is paired with every other drug. X-axis denotes the five other paired drugs separated by a black line and categorized into their respective 4 concentrations in uM (0.01, 0.1, 1, 10).

bioRxiv preprint doi: https://doi.org/10.64898/2026.09.08.749477; this version posted September 10, 2026. The copyright holder for this preprint (which was not certified by peer review) is the author/funder, who has granted bioRxiv a license to display the preprint in perpetuity. It is made available under a CC-BY 4.0 International license.

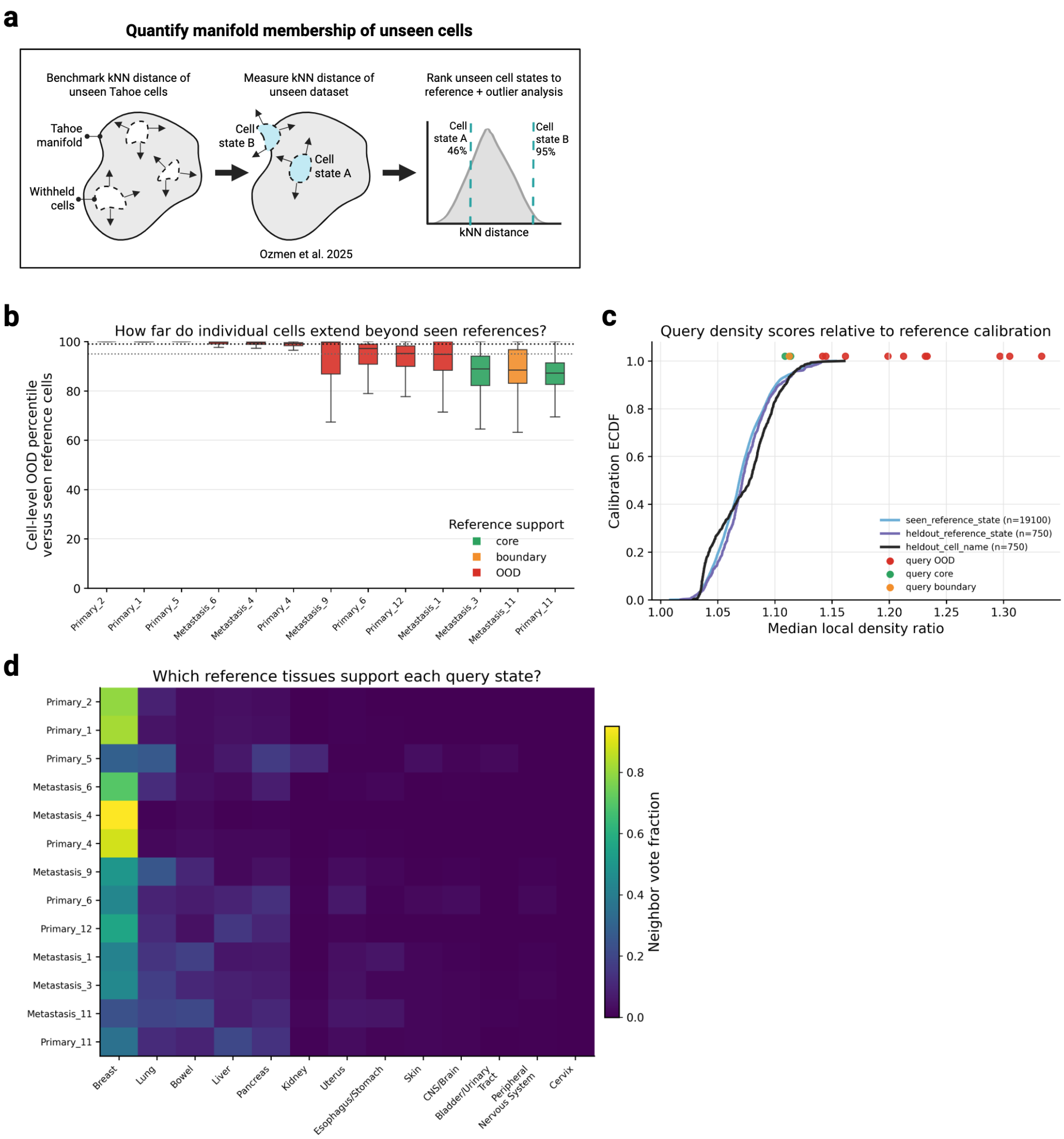

Figure S9: Manifold admissibility of HR+/HER2− breast cancer. a, Part 2 of the admissibility framework. Quantify Tahoe-100M manifold membership of unseen cell states. **b,** Cell-level manifold percentiles relative to withheld Tahoe-100M cells. Query states categorized into core (green), boundary (yellow), and out of distribution (red). **c,** Empirical cumulative distributions show median local-density ratios for seen, withheld-state, and withheld-cell-line Tahoe-100M calibrations. Larger values indicate weaker reference support. Query states are plotted above the distributions and colored by their support classification. **d,** Distance-weighted tissue annotations of the nearest Tahoe-100M reference neighbors for each query state. Rows denote query states, columns denote reference tissues and color indicates the fraction of total neighbor weight assigned to each tissue.

bioRxiv preprint doi: https://doi.org/10.64898/2026.09.08.749477; this version posted September 10, 2026. The copyright holder for this preprint (which was not certified by peer review) is the author/funder, who has granted bioRxiv a license to display the preprint in perpetuity. It is made available under a CC-BY 4.0 International license.

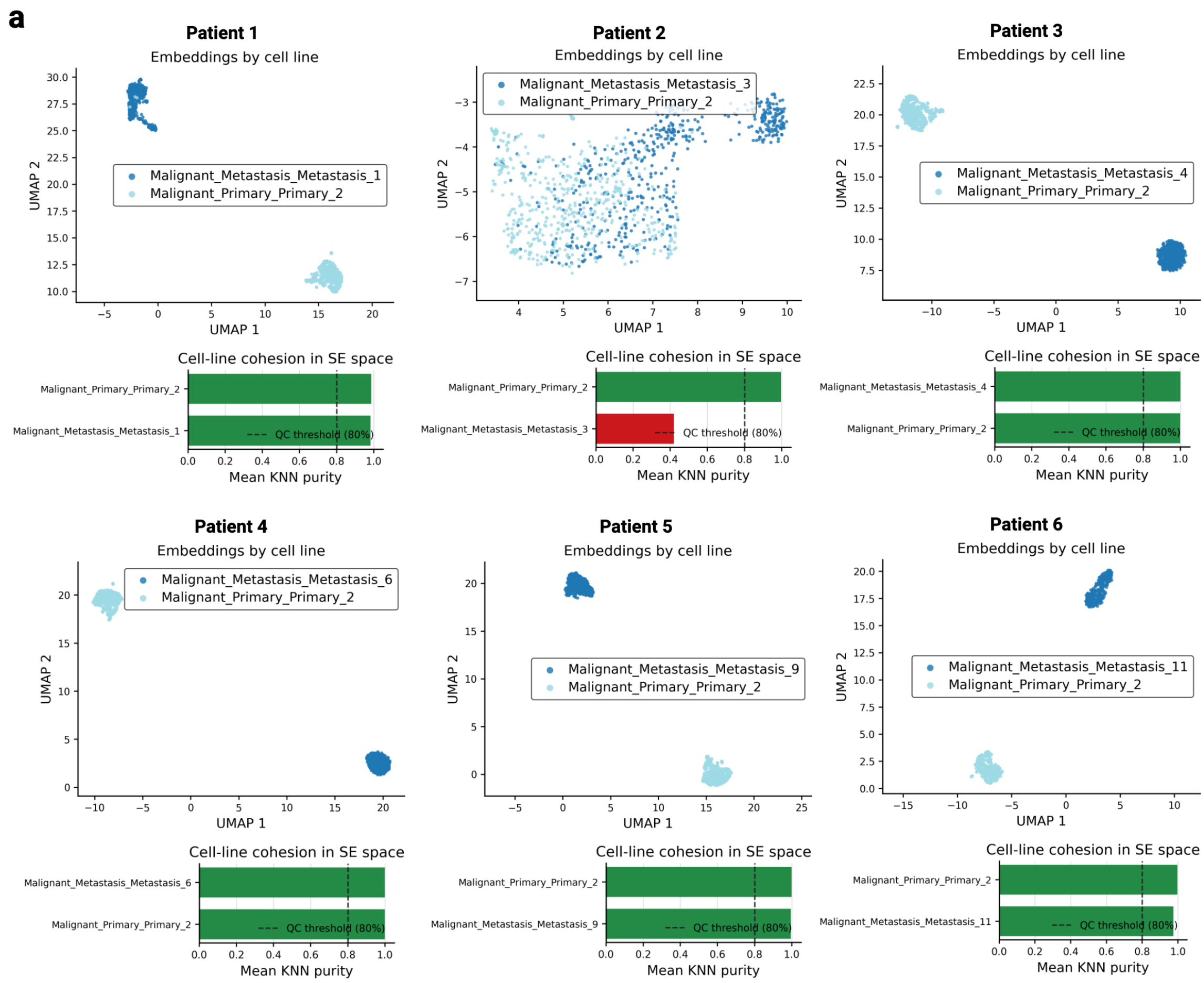

Figure S10: Source-target separation admissibility for all six metastatic-to-primary conversions for HR+/HER2− breast cancer. a, Part 3 of the admissibility framework to define a distinguishable objective for the six patients. Top: UMAP separation of source (metastatic) and target (primary) in STATE single-cell foundation model embedding space. Bottom: barplot of mean KNN purity per cell state. Dotted line indicates a purity threshold of 80%.

bioRxiv preprint doi: https://doi.org/10.64898/2026.09.08.749477; this version posted September 10, 2026. The copyright holder for this preprint (which was not certified by peer review) is the author/funder, who has granted bioRxiv a license to display the preprint in perpetuity. It is made available under a CC-BY 4.0 International license.

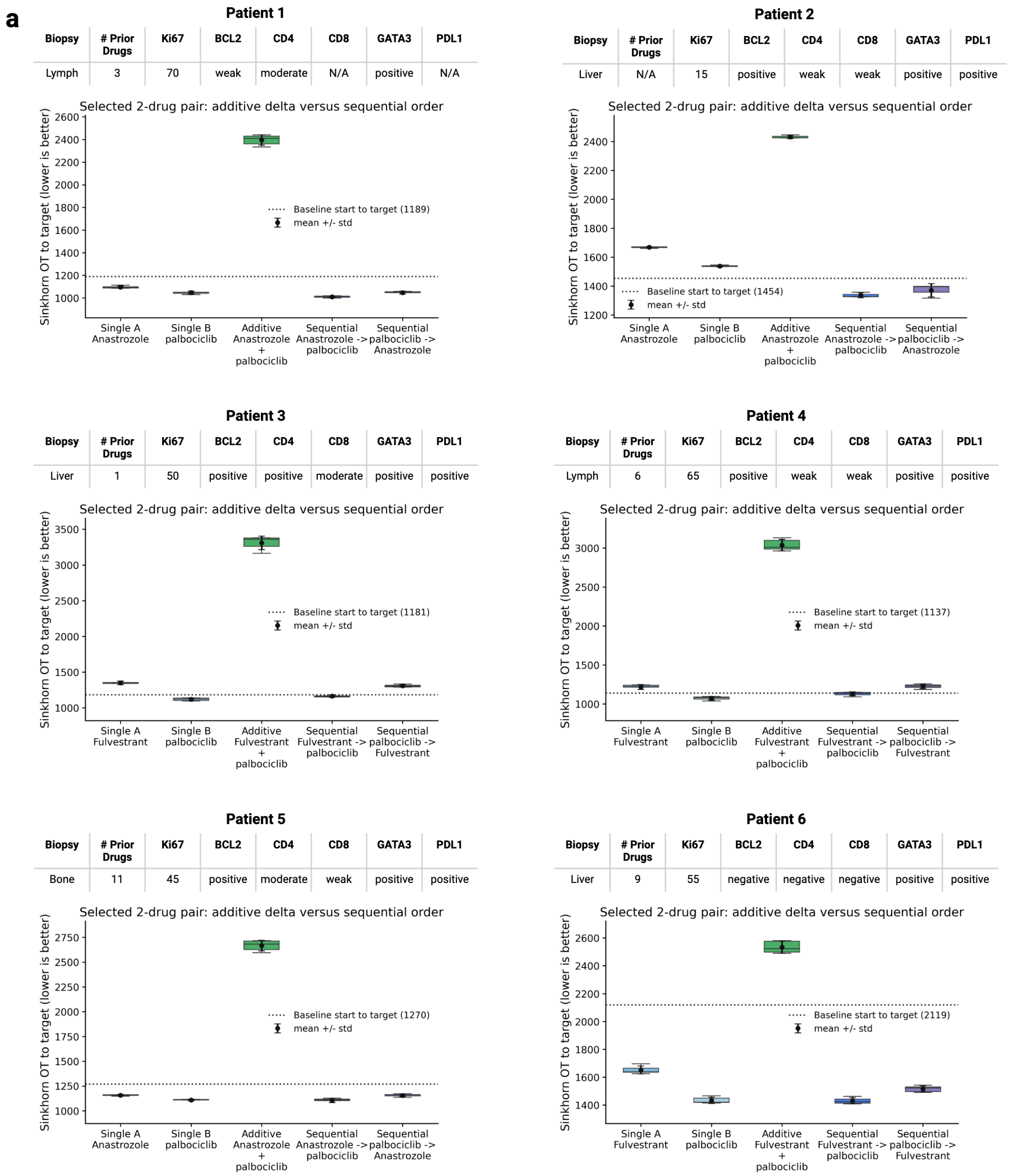

Figure S11: Sequential drug comparisons for all six metastatic-to-primary conversions for HR+/HER2− breast cancer. a, For each patient conversion from source (metastatic) to target (primary), the best 2-drug pair from the 9 FDA approved 2-drug pairs for HR+/HER2− breast cancer was selected. Boxplot of OT distance to target of starting cell state under multiple perturbation conditions of the known 2-drug combination including in order: single drug A, single drug B, additive displacement vectors of individual drug A and B, sequential A then B, sequential B then A.

bioRxiv preprint doi: https://doi.org/10.64898/2026.09.08.749477; this version posted September 10, 2026. The copyright holder for this preprint (which was not certified by peer review) is the author/funder, who has granted bioRxiv a license to display the preprint in perpetuity. It is made available under a CC-BY 4.0 International license.

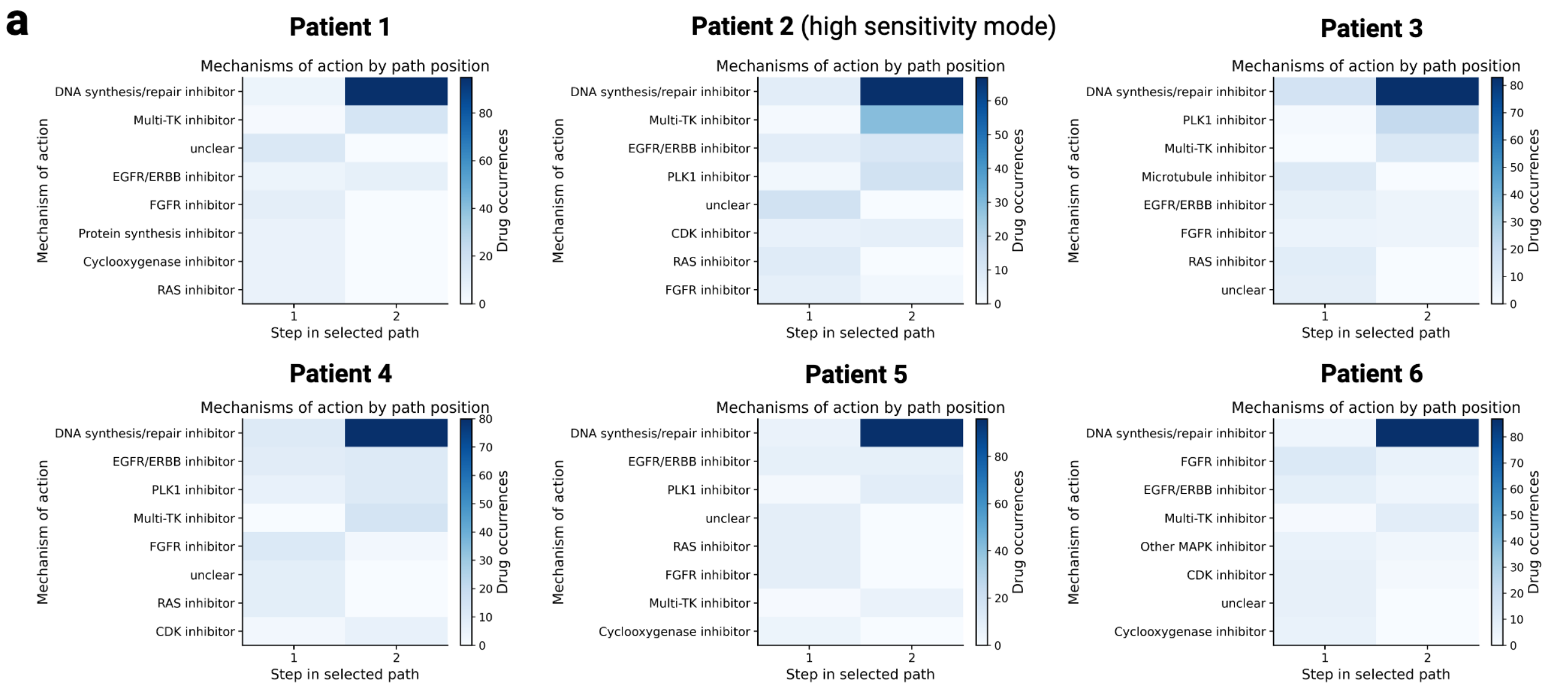

Figure S12: Mechanism of action frequency per patient for the conversion of metastatic malignant cells into primary tumor malignant cells. a, 6 patients with HR+/HER2- metastatic breast cancer. PHAROS open search conversion from metastatic malignant cells to primary malignant cells. Heatmaps, one per patient, of MOA frequencies across each step of the 2-step trajectory for the top 128 paths.

bioRxiv preprint doi: https://doi.org/10.64898/2026.09.08.749477; this version posted September 10, 2026. The copyright holder for this preprint (which was not certified by peer review) is the author/funder, who has granted bioRxiv a license to display the preprint in perpetuity. It is made available under a CC-BY 4.0 International license.

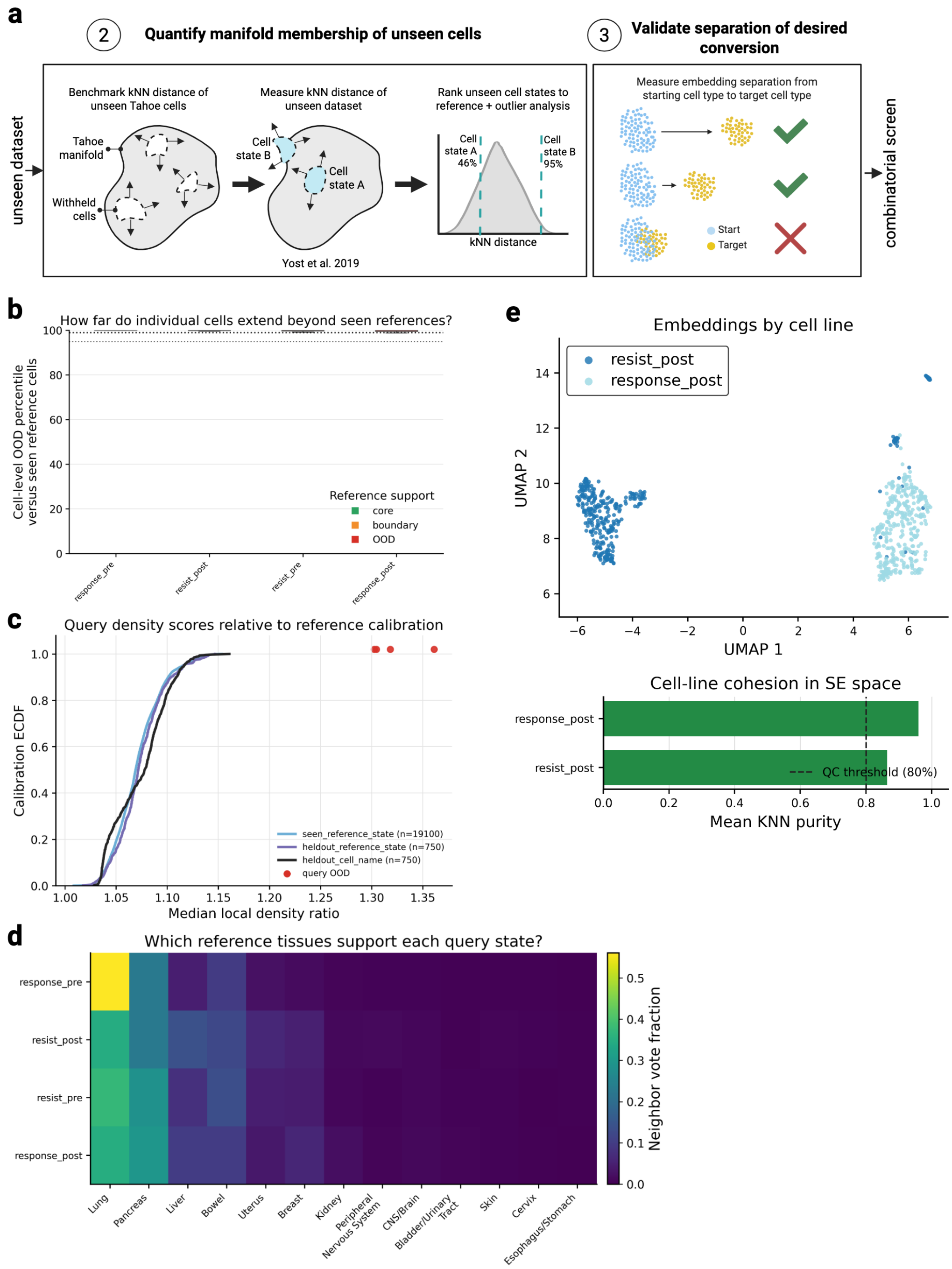

Figure S13: Manifold admissibility of basal cell carcinoma. **a,** Part 2 and 3 of the admissibility framework. **b,** Cell-level manifold percentiles relative to withheld Tahoe-100M cells. Query states categorized into core (green), boundary (yellow), and out of distribution (red). **c,** Empirical cumulative distributions show median local-density ratios for seen, withheld-state, and withheld-cell-line Tahoe-100M calibrations. Larger values indicate weaker reference support. Query states are plotted above the distributions and colored by their support classification. **d,** Distance-weighted tissue annotations of the nearest Tahoe-100M reference neighbors for each query state. Rows denote query states, columns denote reference tissues and color indicates the fraction of total neighbor weight assigned to each tissue. **e,** Top: UMAP separation of source (Post-immunotherapy non-responder malignant cells) and target (Post-immunotherapy responder malignant cells) in STATE single-cell foundation model embedding space. Bottom: barplot of mean KNN purity per cell state. Dotted line indicates a purity threshold of 80%.

bioRxiv preprint doi: https://doi.org/10.64898/2026.09.08.749477; this version posted September 10, 2026. The copyright holder for this preprint (which was not certified by peer review) is the author/funder, who has granted bioRxiv a license to display the preprint in perpetuity. It is made available under a CC-BY 4.0 International license.

S2 Detailed formulation of the PHAROS conversion and search engine

This section provides an algorithmic description of the PHAROS conversion engine. Exact preprocessing, construction of the conversion-aligned PCA–PLS-DA scoring space, and experiment-specific settings are described in Methods Sections 4.5–4.9. Both hypothesis-driven evaluation and open search use the same frozen state-transition and distribution-scoring engine; they differ only in how candidate perturbation paths are generated.

S2.1 Cell-state conversion objective

Let $\mathbf{X}^{(0)} \in \mathbb{R}^{N\times D}$ denote a sampled source population and $\mathbf{Y} \in \mathbb{R}^{M\times D}$ a sampled target population, represented by $\mathrm{STATE}_{\mathrm{SE}}$ embeddings. A perturbation label p specifies a drug and concentration from the Tahoe vocabulary. For an ordered perturbation path

$$\pi = (p_1, \ldots, p_L),$$

PHAROS recursively predicts the cell state after each perturbation as

$$\mathbf{X}^{(t)} = f_{\text{ST-SE}}\left(\mathbf{X}^{(t-1)}, p_t\right), \qquad t = 1, \ldots, L, \tag{S1}$$

where $f_{\text{ST-SE}}$ is the frozen pretrained state-transition model. This differs from an additive displacement approximation, in which single-drug effects are estimated from the same initial state and then summed. Consequently, PHAROS predictions may depend on perturbation order.

Let Π denote the conversion-specific scoring map. In the reported conversion-aligned analyses, Π is the fitted PCA–PLS-DA transformation described in Methods Section 4.5. Importantly, Π is used only for scoring: all ST-SE transitions in Eq. (S1) are computed in the original full SE embedding space. The terminal state produced by path π is scored against the target by entropically regularized Sinkhorn optimal transport:

$$J(\pi) = \mathrm{OT}_{\varepsilon,c}\left(\Pi(\mathbf{X}^{(L)}), \Pi(\mathbf{Y})\right). \tag{S2}$$

S2.2 Candidate generation

The hypothesis-driven and open-search modes use the same objective in Eq. (S2). In hypothesis-driven evaluation, candidate drug pairs are specified in advance. PHAROS evaluates the allowed drug concentrations and both perturbation orders and retains the configuration producing the lowest target distance.

In open search, candidate identities are not specified. PHAROS instead approximates

$$\pi^\star = \arg\min_{\pi\in\mathcal{P}^L} J(\pi) \tag{S3}$$

using diverse beam search, where $\mathcal{P}$ is the searchable set of drug–concentration perturbation labels.

S2.3 Robust diverse beam search

At each search depth, every retained partial path is extended by every legal next perturbation. Candidate states are first scored using a 10-iteration Sinkhorn approximation. The best αB candidates are then recomputed with the full 100-iteration Sinkhorn calculation.

To reduce sensitivity to the particular cells sampled in the source and target populations, candidates surviving this prefilter are replayed across multiple source–target batches. For a candidate path π, let $J_b(\pi)$ denote its Sinkhorn target distance in batch b. PHAROS uses the robust score

$$R(\pi) = \frac{1}{S}\sum_{b=1}^{S} J_b(\pi) + 0.5\,\mathrm{SD}\left\{J_1(\pi), \ldots, J_S(\pi)\right\}. \tag{S4}$$

bioRxiv preprint doi: https://doi.org/10.64898/2026.09.08.749477; this version posted September 10, 2026. The copyright holder for this preprint (which was not certified by peer review) is the author/funder, who has granted bioRxiv a license to display the preprint in perpetuity. It is made available under a CC-BY 4.0 International license.

A standard beam search can become dominated by highly similar paths where drugs used are the same with varying concentrations. For candidate π and the set $\mathcal{B}_{\text{sel}}$ of paths already selected into the new beam, PHAROS defines

$$D(\pi) = R(\pi) + 25 \max_{\pi' \in \mathcal{B}_{\text{sel}}} \text{overlap}(\pi, \pi'), \tag{S5}$$

where $\text{overlap}(\pi, \pi')$ is the fractional overlap in base-drug identities between the two paths. When $\mathcal{B}_{\text{sel}}$ is empty, the overlap penalty is zero. Candidates are added sequentially according to increasing $D(\pi)$ until the beam is full.

For all reported two-drug open searches, the beam width was $B = 128$, the maximum depth was $L_{\text{max}} = 2$, and the prefilter multiplier was $\alpha = 10$.

S2.4 Path constraints

Perturbation labels corresponding to DMSO were excluded from the searchable library. Different concentrations of the same drug are distinct perturbation labels, but once a base drug appears in a path, no other concentration of that drug may be selected later in the same path.

bioRxiv preprint doi: https://doi.org/10.64898/2026.09.08.749477; this version posted September 10, 2026. The copyright holder for this preprint (which was not certified by peer review) is the author/funder, who has granted bioRxiv a license to display the preprint in perpetuity. It is made available under a CC-BY 4.0 International license.

Algorithm 1 PHAROS robust diverse beam search

Input: Source cloud $\mathbf{X}^{(0)}$, target cloud $\mathbf{Y}$, perturbation library $\mathcal{P}$, scoring map Π, beam width B, maximum depth $L_{\max}$, prefilter multiplier α
Output: Retained perturbation paths

$\mathcal{B} \leftarrow \{(\emptyset, \mathbf{X}^{(0)})\}$
for $t = 1$ **to** $L_{\max}$ **do**
  $\mathcal{C} \leftarrow \emptyset$
  for each $(\pi, \mathbf{X}) \in \mathcal{B}$ **do**
    for each legal $p \in \mathcal{P}$ **do**
      $\mathbf{X}' \leftarrow f_{\text{ST-SE}}(\mathbf{X}, p)$
      $s \leftarrow \mathrm{OT}_{10}\,(\Pi(\mathbf{X}'), \Pi(\mathbf{Y}))$
      add $(\pi \| p, \mathbf{X}', s)$ to $\mathcal{C}$
    end for
  end for
  retain the αB candidates in $\mathcal{C}$ with lowest s
  recompute retained candidates using OT_{100}
  for each retained candidate path π **do**
    replay π on each source–target batch b
    compute $J_b(\pi)$ using full Sinkhorn OT
    $R(\pi) \leftarrow \mathrm{mean}_b\, J_b(\pi) + 0.5\, \mathrm{SD}_b\, J_b(\pi)$
  end for
  $\mathcal{B}_{\text{sel}} \leftarrow \emptyset$
  while $|\mathcal{B}_{\text{sel}}| < B$ **do**
    for each remaining candidate π **do**
      $D(\pi) \leftarrow R(\pi) + 25 \max_{\pi' \in \mathcal{B}_{\text{sel}}} \mathrm{overlap}(\pi, \pi')$ ▷ penalty $= 0$ if $\mathcal{B}_{\text{sel}} = \emptyset$
    end for
    add candidate with minimum $D(\pi)$ to $\mathcal{B}_{\text{sel}}$
  end while
  $\mathcal{B} \leftarrow \mathcal{B}_{\text{sel}}$
end for
return $\mathcal{B}$

Here $\pi \| p$ denotes appending perturbation p to path π. Drug–concentration labels are treated as distinct actions during expansion, whereas path-reuse constraints and the diversity penalty operate on base-drug identity.

bioRxiv preprint doi: https://doi.org/10.64898/2026.09.08.749477; this version posted September 10, 2026. The copyright holder for this preprint (which was not certified by peer review) is the author/funder, who has granted bioRxiv a license to display the preprint in perpetuity. It is made available under a CC-BY 4.0 International license.

S3 Model validation - interpreting the model given our framework

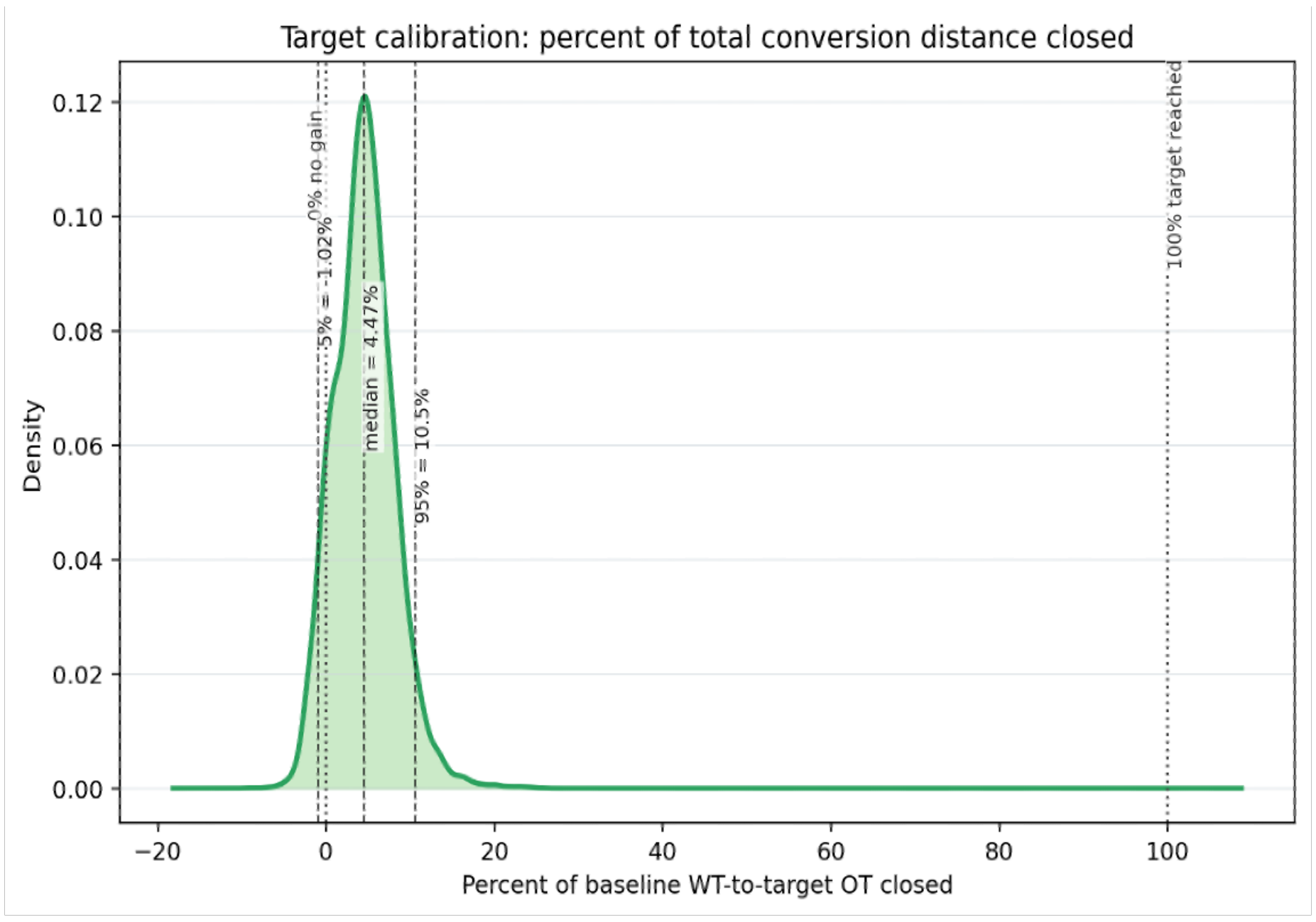

Figure S14: Target calibration in full-d space. Density plot of the percentage of the initial WT-to-target optimal-transport (OT) distance (in full-d space) closed when a group of WT cells is passed through the model with the ground-truth drug as the perturbation label. Each observation corresponds to a combination of drug and cell-line across Tahoe, with the target being the true perturbed population for that combination. Read S4 to understand better the difference in results between the compressed and the full-d space.

bioRxiv preprint doi: https://doi.org/10.64898/2026.09.08.749477; this version posted September 10, 2026. The copyright holder for this preprint (which was not certified by peer review) is the author/funder, who has granted bioRxiv a license to display the preprint in perpetuity. It is made available under a CC-BY 4.0 International license.

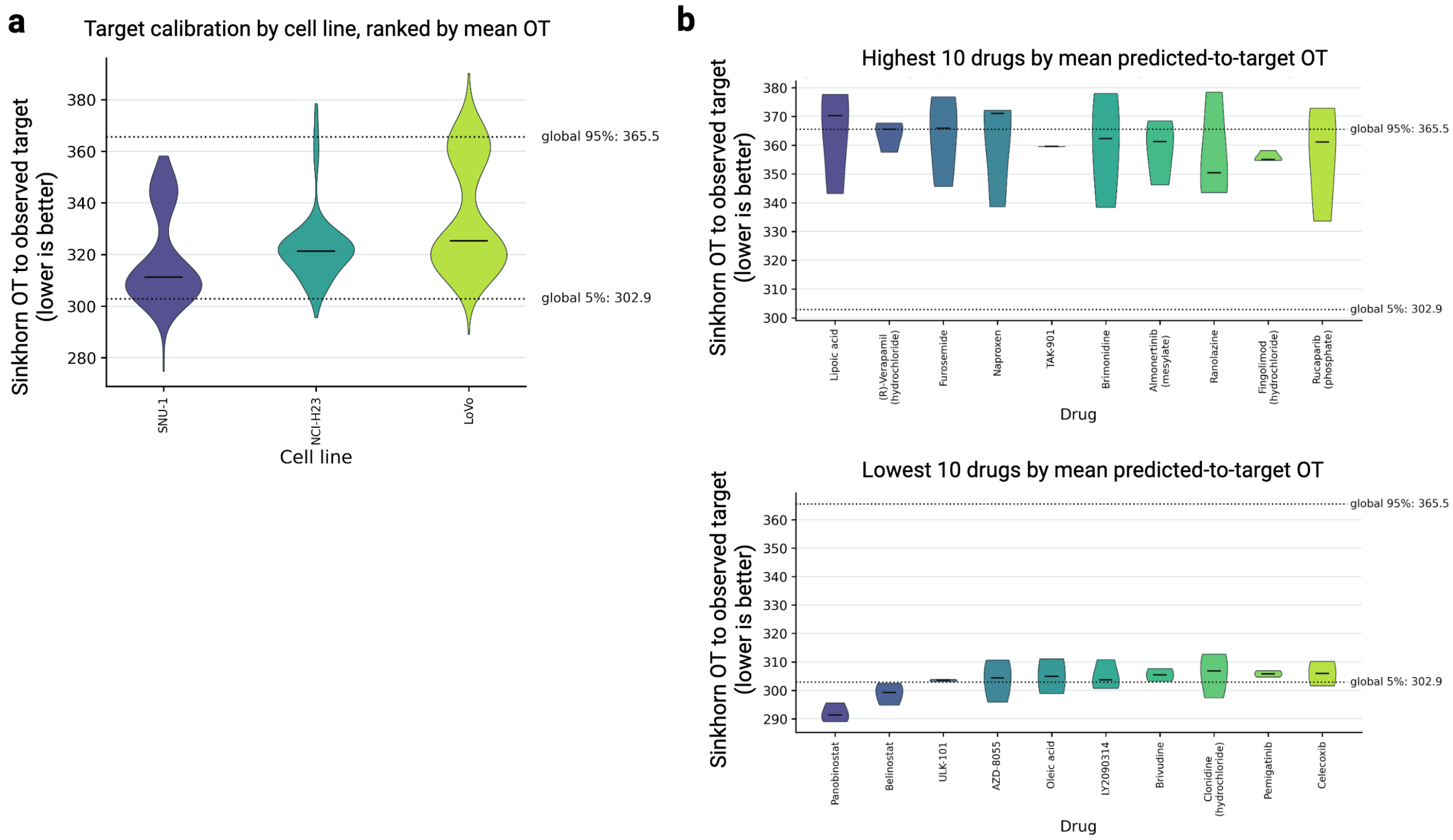

Figure S15: Target calibration by cell line and drug. a, Density plots of OT distance between a group of WT cells passed through the model with the ground-truth drug as the perturbation label and a group of experimental cells perturbed with the same drug. Each observation corresponds to a combination of drug and cell-line across Tahoe, and the data is grouped by cell-line. **b,** Same as in (a) but grouped by drug and showing only highest and lowest drugs by mean OT distance predicted-to-target.

bioRxiv preprint doi: https://doi.org/10.64898/2026.09.08.749477; this version posted September 10, 2026. The copyright holder for this preprint (which was not certified by peer review) is the author/funder, who has granted bioRxiv a license to display the preprint in perpetuity. It is made available under a CC-BY 4.0 International license.

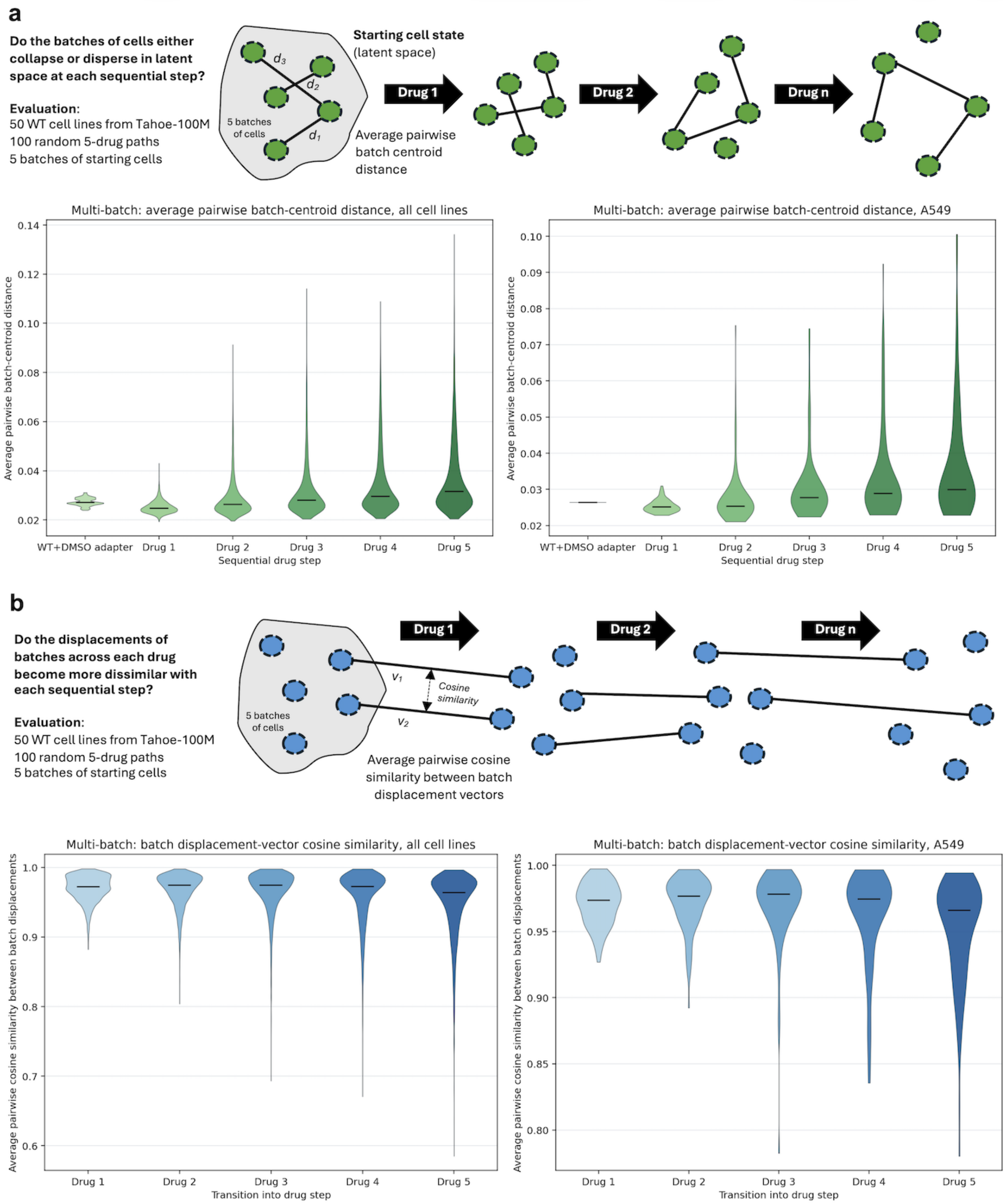

Figure S16: Stability of sequential ST-SE rollouts across drug steps. a-b, Evaluated over all 50 WT (no drug) cell lines from Tahoe-100M across 5 batches of starting cell states and 100 random 5 drug step trajectories. **a,** Quantifying how the distribution of batches evolves at every step. Distribution of average pairwise batch-centroid distance at each step of the 5 drug trajectories. Left: all cell lines. Right: A549. **b,** Quantifying how similar the batches are displaced with each sequential drug. Distribution of average pairwise cosine similarity between batch displacements comparing cell states in drug N and drug N-1. Left: all cell lines. Right: A549.

bioRxiv preprint doi: https://doi.org/10.64898/2026.09.08.749477; this version posted September 10, 2026. The copyright holder for this preprint (which was not certified by peer review) is the author/funder, who has granted bioRxiv a license to display the preprint in perpetuity. It is made available under a CC-BY 4.0 International license.

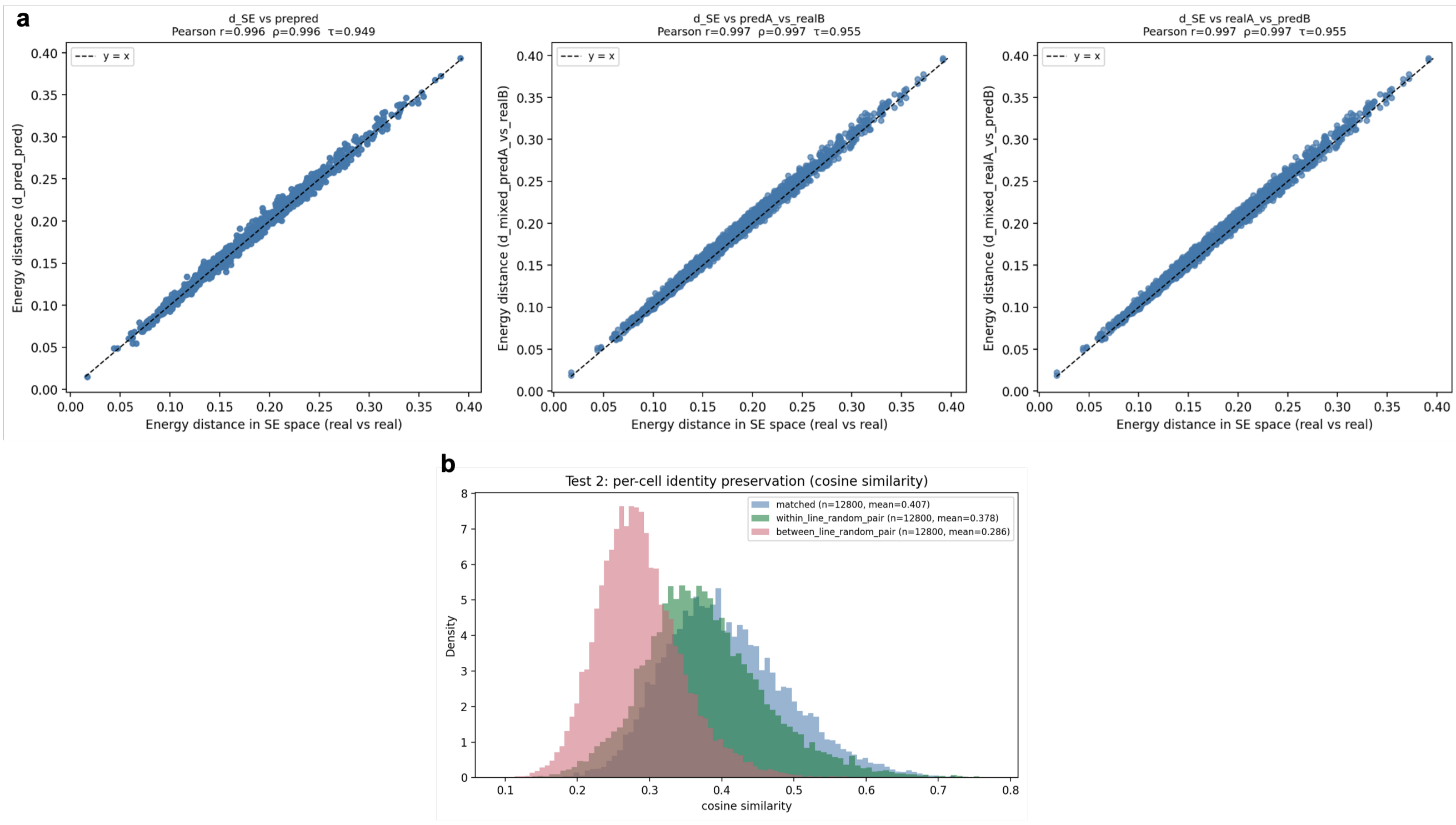

Figure S17: Alignment for unperturbed cells between SE and ST-SE. a, The first scatter plot shows the correlation between the energy distance of two groups of 256 unperturbed cells from different cell lines computed in SE space and the same metric in ST-SE space, that is after the DMSO adapter. In the other two scatter plots only one of the two groups is passed through the ST-SE model (DMSO adapter), while the other is kept in SE space. **b,** The overlaid density histogram compare the distribution of cosine similarity across three groups. In the matched group, for each cell we compute the cosine similarity between its unperturbed SE embedding and the ST-SE output for the same cell after passing it through the predictor with DMSO as the perturbation label. In the within-line random pair group, we compute cosine similarity between SE embeddings of two different unperturbed cells sampled from the same cell line (neither passed through ST-SE). In the between-line random pair group, we compute cosine similarity between the SE embedding of a cell in one line and the SE embedding of a cell drawn from a different cell line.

bioRxiv preprint doi: https://doi.org/10.64898/2026.09.08.749477; this version posted September 10, 2026. The copyright holder for this preprint (which was not certified by peer review) is the author/funder, who has granted bioRxiv a license to display the preprint in perpetuity. It is made available under a CC-BY 4.0 International license.

S4 Understanding the effect of dimensionality reduction

In this section, an informal noise model is presented, which serves to motivate the use of dimensionality reduction techniques, such as PCA– PLS-DA, to enhance the match between the predicted and target cell populations. This provides a theoretical explanation for the differences observed between the compressed and full-dimensional spaces. The Sinkhorn optimal-transport distance metric is employed for the analysis, with a primary focus on the cost matrix.

Consider two empirical cell populations

$$X = \{x_i\}_{i=1}^{N}, \qquad Y = \{y_j\}_{j=1}^{M},$$

where each cell embedding is decomposed into a biologically informative component and a nuisance component:

$$x_i = (x_i^S, x_i^Z), \qquad y_j = (y_j^S, y_j^Z).$$
$$x_i^S, y_j^S \in \mathbb{R}^k, \qquad x_i^Z, y_j^Z \in \mathbb{R}^m,$$

with $m = D - k$. We assume that the nuisance coordinates have the same distribution in the two populations,

$$x_i^Z \sim P_Z, \qquad y_j^Z \sim P_Z,$$

thus do not contain useful information for distinguishing the two states.

Using the squared Euclidean cost, the pairwise cost between cells x_i and y_j is

$$C_{ij} = \|x_i - y_j\|_2^2 = \|x_i^S - y_j^S\|_2^2 + \|x_i^Z - y_j^Z\|_2^2.$$

We define

$$A_{ij} = \|x_i^S - y_j^S\|_2^2, \qquad B_{ij} = \|x_i^Z - y_j^Z\|_2^2,$$

so that

$$C_{ij} = A_{ij} + B_{ij}.$$

A_{ij} is the biologically relevant contribution to the cost, whereas B_{ij} is induced by nuisance variation.

Assume that the nuisance coordinates are independent and have equal variance σ^2. For a single nuisance coordinate, define

$$d_\ell = x_{i,\ell}^Z - y_{j,\ell}^Z.$$

Since $x_{i,\ell}^Z$ and $y_{j,\ell}^Z$ are independent and identically distributed,

$$\mathbb{E}[d_\ell] = 0, \qquad \mathrm{Var}(d_\ell) = 2\sigma^2.$$

Therefore,

$$\mathbb{E}[B_{ij}] = \sum_{\ell=1}^{m} \mathbb{E}[d_\ell^2] = 2m\sigma^2.$$

If the nuisance coordinates are Gaussian, then

$$d_\ell \sim \mathcal{N}(0, 2\sigma^2),$$

and hence

$$\mathrm{Var}(d_\ell^2) = 8\sigma^4.$$

It follows that

$$\mathrm{Var}(B_{ij}) = \sum_{\ell=1}^{m} \mathrm{Var}(d_\ell^2) = 8m\sigma^4,$$

and

$$\mathrm{sd}(B_{ij}) = \sqrt{8m}\sigma^2.$$

bioRxiv preprint doi: https://doi.org/10.64898/2026.09.08.749477; this version posted September 10, 2026. The copyright holder for this preprint (which was not certified by peer review) is the author/funder, who has granted bioRxiv a license to display the preprint in perpetuity. It is made available under a CC-BY 4.0 International license.

Thus the nuisance contribution can be decomposed as

$$B_{ij} = 2m\sigma^2 + \delta_{ij},$$

where

$$\mathbb{E}[\delta_{ij}] = 0, \qquad \mathrm{sd}(\delta_{ij}) = \sqrt{8m}\sigma^2.$$

While the constant term $2m\sigma^2$ shifts all pairwise costs by the same amount, the problematic component is the fluctuation δ_{ij}, because it can change the relative ordering of the entries of the cost matrix.

Informally, for population matching to be driven by biological structure rather than nuisance variation, differences in signal costs should dominate nuisance fluctuations. For two candidate cell pairs (i, j) and (i', j'), this requires

$$|A_{ij} - A_{i'j'}| \gg |\delta_{ij} - \delta_{i'j'}|.$$

Since the scale of δ_{ij} is

$$\mathrm{sd}(\delta_{ij}) = \sqrt{8m}\sigma^2,$$

a useful informal signal-to-noise ratio is

$$\mathrm{SNR}_{\mathrm{cost}} \approx \frac{\text{typical variation in } A_{ij}}{\sqrt{m}\sigma^2}.$$

As the number of nuisance dimensions m increases, this ratio decreases, and a low SNR can make pairwise costs noisy and distort population matching.

Since PCA–PLS-DA reduces dimensionality by selecting directions that best discriminate between the two populations, it can significantly reduce nuisance variation while preserving signal associated with the population-level contrast. This motivates computing Sinkhorn OT scores after applying a supervised dimensionality reduction step, especially when embeddings contain lots of dimensions that are weakly informative for the conversion of interest.

S5 CPA vs STATE

We also considered using CPA within PHAROS as the perturbation predictor, primarily due to the model structure, which, at least theoretically, allows drugs to be applied simultaneously in latent space, thereby eliminating the sequential application strategy required by our ST-SE implementation. Furthermore, to our knowledge, CPA is the only model that can be effectively applied to predict multiple drug perturbations with single-cell resolution. However, we encountered significant challenges in identifying a solution to two substantial limitations. Firstly, CPA lacks the capacity to regulate dataset-specific effects, a capacity that is partially addressed by STATE via the utilization of the DS token in the SE module. Secondly, in its standard formulation, CPA does not possess the capability to predict the effects of perturbations in unseen cell lines because cell-line identity is encoded via categorical covariate embeddings that are undefined at inference for unseen lines. To solve such limitations one could modify the architecture to eliminate both cell line embeddings and the adversarial task of predicting cell lines, and train using SE embeddings both for the model's input and output spaces. However, this would significantly deviate from the original CPA architecture and demand large-scale retraining on the whole Tahoe corpus, whereas PHAROS relies on a pretrained ST-SE model.

S6 Interpretation of partial positive-control recovery

In some positive controls, the experimentally applied drug pair does not lie in the lower tail of the OT-distance-to-target distribution of random two-drug perturbations. Several non-exclusive explanations may account for this. First, ST-SE is far from a perfect perturbation predictor. Error compounds as we predict sequentially, especially when at the intermediate step the cell moves to an out-of-distribution part of the space. Furthermore PHAROS applies drugs sequentially ($A \to B$), whereas positive controls are based on simultaneous co-treatment ($A + B$). In certain scenarios order may matter, that is $A \to B$

bioRxiv preprint doi: https://doi.org/10.64898/2026.09.08.749477; this version posted September 10, 2026. The copyright holder for this preprint (which was not certified by peer review) is the author/funder, who has granted bioRxiv a license to display the preprint in perpetuity. It is made available under a CC-BY 4.0 International license.

(and/or $B \to A$) is substantially different from $A + B$. For example, this might be the case if two drugs inhibit parallel, partially redundant branches of a regulatory network. Lastly, PHAROS might struggle even more when dealing with drug combinations characterized by synergistic or antagonistic behaviors, that is combinations whose joint effect deviates from the addition of single-drug responses. Importantly, at least in theory, a sequential predictive approach might help in this case as the second drug is applied conditional on the cell cloud produced by the first. This advantage assumes that the intermediate state is predicted accurately and remains in-distribution for ST-SE.

S7 Proposed reinforcement-learning search (not evaluated)

Beam search was used for all experiments reported in this study. The major disadvantage is that such an algorithm is greedy with respect to the distance metric at each step and can miss paths requiring temporarily suboptimal intermediate perturbations that position the cloud embeddings in a favorable part of the space for later moves. To tackle this problem, drug-path discovery can be formulated as a finite-horizon Markov decision process (MDP) in which the pretrained ST-SE model defines the transition dynamics. We outline this formulation for completeness, but it was not implemented.

S7.1 MDP formulation

We model sequential drug selection as an MDP $(\mathcal{S}, \mathcal{A}, \mathcal{P}, \mathcal{R}, \gamma)$.

Environment state. At step t, the environment maintains a cell cloud $\mathbf{X}_t \in \mathbb{R}^{N \times D}$. A fixed target cloud $\mathbf{Y} \in \mathbb{R}^{M \times D}$ is sampled once per episode from a target state. The starting cloud $\mathbf{X}_0$ is sampled from the starting state.

Actions. An action $a_t \in \mathcal{A}$ is a perturbation label (drug and concentration) from the ST-SE vocabulary. The same path constraints as in Section S2.4 apply.

Transitions. Transitions are determined by the sequential application of ST-SE:

$$\mathbf{X}_{t+1} = f_{\text{ST-SE}}(\mathbf{X}_t, a_t), \tag{S6}$$

as in Eq. (??). Given $\mathbf{X}_t$ and a_t, the next state is deterministic. Episodes have maximum length $L_{\max}$.

Reward. We define a reward function using Sinkhorn optimal-transport (OT) distance to the target cloud $\mathbf{Y}$:

$$r_t = \frac{\mathrm{OT}(\mathbf{X}_t, \mathbf{Y}) - \mathrm{OT}(\mathbf{X}_{t+1}, \mathbf{Y})}{\mathrm{OT}(\mathbf{X}_0, \mathbf{Y})}. \tag{S7}$$

Positive reward therefore corresponds to progress toward the target distribution.

S7.2 Goal-conditioned Q-network

We propose a single action-value network $Q(\mathbf{s}_t, a; \boldsymbol{\theta})$ trained across many start–target pairs. The model is goal-conditioned, that is the target enters the state, allowing for inference on any start–target couple of interest.

State encoding. Because the Q-network requires a fixed-size input, the full cell cloud $\mathbf{X}_t$ is summarized by a map $\psi(\cdot)$ applied to the current and target clouds. For example,

$$\psi(\mathbf{X}) = [\boldsymbol{\mu}(\mathbf{X}),\ \boldsymbol{\sigma}(\mathbf{X})],$$

where $\boldsymbol{\mu}$ and $\boldsymbol{\sigma}$ are the mean and standard deviation (square roots of the diagonal entries of the empirical covariance matrix) across cells. Optionally, ψ can be computed after a fixed global dimensionality reduction of SE embeddings (e.g. PCA fit on all Tahoe cells embedded with SE).

bioRxiv preprint doi: https://doi.org/10.64898/2026.09.08.749477; this version posted September 10, 2026. The copyright holder for this preprint (which was not certified by peer review) is the author/funder, who has granted bioRxiv a license to display the preprint in perpetuity. It is made available under a CC-BY 4.0 International license.

Let $\mathbf{h}_t = \psi(\mathbf{X}_t)$ and $\mathbf{g} = \psi(\mathbf{Y})$ (computed once per episode). The input to the Q-network at step t is

$$\mathbf{s}_t = [\mathbf{h}_t,\ \mathbf{g},\ \boldsymbol{\tau}_t], \tag{S8}$$

where $\boldsymbol{\tau}_t = \mathbf{e}_{L_{\max}-t} \in \{0,1\}^{L_{\max}+1}$, $\mathbf{e}_k$ is the kth standard basis vector.

Learning. Following the deep Q-network (DQN) framework, we approximate $Q^*(\mathbf{s}, a)$ with a neural network $Q(\mathbf{s}_t, a; \boldsymbol{\theta})$ optimized by temporal-difference learning. Illegal actions are masked when computing $\max_{a'} Q(\mathbf{s}_{t+1}, a'; \boldsymbol{\theta}^-)$. A periodically updated target network $\boldsymbol{\theta}^-$ stabilizes training. As with standard function approximation, convergence to Q^* is not guaranteed.

Inference. At inference, given a starting and a target state of interest, the trained action-value network $Q(\mathbf{s}_t, a; \boldsymbol{\theta})$ constructs a predicted path by choosing $a_t = \arg\max_{a \in \mathcal{A}_{\text{legal}}(\mathbf{s}_t)} Q(\mathbf{s}_t, a; \boldsymbol{\theta})$ at each step and updating $\mathbf{X}_{t+1} = f_{\text{ST-SE}}(\mathbf{X}_t, a_t)$. Alternatively, the trained neural network can be employed within a tree-search procedure (e.g. beam search) to search for additional promising paths.

S7.3 Training data (proposed)

Learning requires a diverse set of simulated experiences sampled within the Tahoe manifold, otherwise ST-SE would have to predict out-of-distribution transitions. In practice, one would select a collection of start–target cell-line pairs whose SE embeddings span this space in a representative way. Episodes would then be generated by rolling out ST-SE from sampled starting clouds toward fixed target clouds, with OT-based rewards as in Eq. (S7), and actions chosen ϵ-greedily: with probability ϵ a legal perturbation is sampled uniformly at random, and with probability $1-\epsilon$ the greedy action $\arg\max_{a \in \mathcal{A}_{\text{legal}}(\mathbf{s}_t)} Q(\mathbf{s}_t, a; \boldsymbol{\theta})$ is chosen. We do not specify a particular pair-selection, but the main requirement is coverage and diversity of (c_s, c_t) tasks within the Tahoe manifold.

S7.4 Relation to beam search

Both approaches use the same ST-SE transition model and OT-based notion of progress toward a target distribution. Beam search evaluates explicit multi-step paths without learning and was used for all results in this study. Reinforcement learning could in principle yield a reusable policy across conversion tasks, but each environment step requires a full ST-SE forward pass on N cells, and training would require many simulated episodes. We therefore leave this as a direction for future work.
